# Phillyrin Modulates AMPK‐Associated Cellular Responses and Migration in PC3 Prostate Cancer Cells

**DOI:** 10.1155/jt/2022957

**Published:** 2026-04-11

**Authors:** Cheng-Hsin Lu, Chun-Hsien Wu, Pei-Fang Hsieh, Richard Chen-Yu Wu, Hsing-Chia Mai, Wei-Lun Huang, Sih-Han Chen, Chien-Ming Lai, Victor C. Lin, Chiang-Ting Wang

**Affiliations:** ^1^ Division of Urology, Penghu Hospital, Magong, Penghu, Taiwan; ^2^ Division of Urology, Department of Surgery, E-Da Hospital, Kaohsiung, Taiwan, edah.org.tw; ^3^ Department of Chemical Engineering and Institute of Biotechnology and Chemical Engineering, I-Shou University, Kaohsiung, Taiwan, isu.edu.tw; ^4^ Department of Nursing, I-Shou University, Kaohsiung, Taiwan, isu.edu.tw; ^5^ Department of Medical Laboratory Science and Biotechnology, Chung-Hwa University of Medical Technology, Tainan, Taiwan; ^6^ School of Medicine, College of Medicine, I-Shou University, Kaohsiung, Taiwan, isu.edu.tw; ^7^ Division of Urology, Department of Surgery, E-Da Cancer Hospital, Kaohsiung, Taiwan, isu.edu.tw; ^8^ Division of Urology, Department of Surgery, Kaohsiung Armed Forces General Hospital, Kaohsiung, Taiwan; ^9^ Division of Urology, Gangshan Branch, Kaohsiung Armed Forces General Hospital, Kaohsiung, Taiwan

**Keywords:** adenosine monophosphate–activated protein kinase, epithelial–mesenchymal transition, phillyrin, prostate cancer

## Abstract

Males with prostate cancer exhibit substantial mortality and metastasis; however, few effective treatment strategies are available for advanced prostate cancer. Phillyrins (PHNs) are lignan glycosides that have been reported to exhibit diverse biological activities, including potential anticancer effects. We aimed (1) to assess the effect of PHN on PC3 prostate cancer cells and (2) to examine cellular responses associated with its activity. To assess the effects of PHN, it was exposed to various concentrations of PHN (1, 2.5, and 5 μM) for 24 h. A wound‐healing assay was performed to evaluate cell migration. Western blotting and immunofluorescence were used to investigate the expression of adenosine monophosphate–activated protein kinase (AMPK)–associated proteins and transcription factors involved in epithelial–mesenchymal transition (EMT). PHN exposure was associated with reduced migratory capacity of PC3 cells under noncytotoxic conditions. PHN exposure was associated with decreased α‐smooth muscle actin, Snail, and Slug expression while increasing E‐cadherin expression. Sirtuin 1 (SIRT1) and nuclear respiratory factor 1 (NRF1) protein levels were upregulated in PHN‐treated cells, accompanied by changes in AMPK protein expression. Pharmacological inhibition with Compound C attenuated PHN‐associated changes in EMT‐related marker expression in PC3 cells. Thus, PHN exposure may influence EMT‐associated phenotypes in PC3 cells, potentially involving AMPK‐associated signaling.

## 1. Introduction

In 2022, prostate cancer (PCa) was the most frequently diagnosed cancer in males in the United States. It is the second leading cause of cancer‐related deaths across all age categories [1]. In the past 10 years, the incidence of PCa has increased across several nations, particularly Asia [2]. In Taiwan, as of 2020, PCa was the fifth most common cancer and the sixth leading cause of cancer‐related mortality in males [3]. Asian patients diagnosed with PCa are more likely to have advanced clinical stages compared to Western populations [4]. While androgen deprivation therapy (ADT) is effective in early‐stage PCa, its therapeutic benefit diminishes as the disease progresses to castration‐resistant PCa [4]. Thus, improving our understanding of the cellular responses and molecular processes associated with PCa progression remains an important research objective.

Given the metabolic rewiring and EMT‐driven migration characteristics of advanced PCa, compounds associated with metabolic and stress‐related signaling pathways, including adenosine monophosphate–activated protein kinase (AMPK)‐related components, are of interest. Phillyrin (PHN), a lignan glycoside from *Forsythia suspensa*, has been reported to be associated with alterations in AMPK‐related signaling and mitochondrial‐related markers in several cell types. These reported properties are relevant to cellular processes implicated in PCa progression, including metabolic stress responses and EMT‐associated phenotypes, providing a rationale to test PHN in PC3 cells as a castration‐resistant model. *F. suspensa* (Thunb.) Vahl (Oleaceae) is a popular Chinese herb traditionally used to treat pyrexia and infections. The extract of *F. suspensa* showed anticancer effects on esophageal carcinoma cells (TE‐13), lung cancer cells (A549), and renal cell carcinoma (786‐0) [5–7]. PHN is a major component. PHN possesses notable biological activities, including anti‐inflammatory, antioxidant, and antiviral properties [8]. It has also been reported to exhibit anticancer‐related effects in experimental models. Wang et al. reported that PHN, in combination with an autophagy inhibitor, was associated with altered cellular responses in laryngeal squamous cell carcinoma models [9]. However, reports examining the cellular effects of PHN in PCa remain limited.

The effects of PHN have been reported to be associated with changes in AMPK‐related signaling and mitochondrial‐related markers in several experimental models [10–14]. Mitochondrial biogenesis, defined as the growth and division of preexisting mitochondria, has been implicated in cancer progression and metastasis [15]. Sulforaphane, a compound present in cruciferous vegetables, is an antioxidant and has been reported to influence mitochondrial biogenesis–associated cell death in PCa cells [16]. Several intracellular pathways have been associated with mitochondrial biogenesis, including the peroxisome proliferator–activated receptor gamma coactivator 1‐alpha (PGC‐1α) family of co‐activators, nuclear respiratory factor 1 (NRF‐1), and the AMPK‐related pathway [17]. As a key factor involved in redox hemostasis and regulating ROS, AMPK plays an important role in cellular response to energy stress. AMPK has been reported to interact with PGC‐1α and to be associated with mitochondrial‐related processes, including mitochondrial dynamics and quality control [18, 19]. Penfold et al. reported that alterations in AMPK‐related signaling in PCa cells were associated with changes in PGC‐1*α* expression and mitochondrial‐related markers [19]. Other factors, such as sirtuin 1 (SIRT1) and NRF1, have been implicated in mitochondrial‐related and energy metabolism–associated processes [20, 21]. Owing to their roles in metabolism and cell survival, targeting mitochondria and AMPK‐associated pathways have attracted interest in cancer‐related research. While AMPK can exert context‐dependent effects in cancer, several studies in PCa have reported associations between AMPK‐related signaling and reduced EMT‐associated migration. We therefore sought to examine whether PHN exposure is associated with alterations in AMPK‐related signaling components and EMT‐associated migratory phenotypes in PC3 cells.

Because of its high energy demand, epithelial–mesenchymal transition (EMT) is sensitive to various energy stress conditions. AMPK, an intracellular energy sensor, has been reported to be associated with EMT‐related processes. According to Liang et al., AMPK expression has been reported to show a positive association with epithelial‐specific markers and a negative association with mesenchymal‐specific markers. EMT has been reported to be associated with reduced AMPK gene expression [22]. Researchers have explored the modulation of AMPK‐associated signaling as a potential approach in cancer‐related studies. Previous studies have reported associations between AMPK‐related signaling and alterations in autophagy as well as EMT‐associated transcription factor expression in PCa [23]. Metformin, a widely used antidiabetic agent that has been linked to AMPK‐related signaling, has been associated with improved overall and cancer‐specific survival in patients with PCa [24]. A pooled analysis of the COU‐AA‐301 and COU‐AA‐302 studies reported metformin use in patients with metastatic castration‐resistant PCa was associated with longer overall survival and a higher prostate‐specific antigen response rate, particularly in combination with abiraterone [25]. Collectively, these findings suggest an association between AMPK‐related signaling and modulation of PCa progression and EMT‐associated phenotypes.

To date, limited studies have examined the cellular effects of PHN in PCa. We sought to examine the effects of PHN exposure on PC3 cell migration and its association with mitochondrial‐related markers. PC3 cells are widely used in PCa research due to their higher metastatic potential compared to DU145 and LNCaP cells. PC3 cells are androgen‐independent and serve as a model for castration‐resistant tumors, enabling investigation into biochemical and cellular changes associated with advanced PCa cells [26]. This study provides initial data characterizing PHN‐associated cellular responses in advanced PCa cells, which may inform future toxicological and translational investigations.

## 2. Material and Methods

### 2.1. Reagent

Compound C (Comp C) and PHN (C_27_H_34_O_11_, purity ≥ 98%) were obtained from Sigma‐Aldrich Chemical in St. Louis, Missouri, USA. Comp C was dissolved in dimethyl sulfoxide and maintained at −20°C.

### 2.2. Cell Environment

PC3 cells, a line derived from bone metastasis, are androgen‐independent and exhibit highly aggressive behavior. They represent castration‐resistant tumors [26]. The PC3 cell line used in this study was obtained from the American Type Culture Collection (Rockville, MD, USA). PC3 cells were cultured in RPMI 1640 medium (Gibco, Grand Island, NY, USA) supplemented with 10% fetal bovine serum (FBS; Gibco, Grand Island, NY, USA) and 1% penicillin–streptomycin (Gibco, Grand Island, NY, USA). Cells were grown at 37°C with 95% air and 5% carbon dioxide. To study the effects of PHN on PC3 cells, the cells were separated into control and different PHN concentration groups. Cells in the experimental groups were exposed to various doses of PHN (1, 2.5, or 5 μM) for 24 h. Cells in the control group were cultivated in a standard culture medium without treatment.

### 2.3. CCK8 Assay

CCK8 (cat. No. ab228554; Abcam, Cambridge, UK) was used to assess cell viability. Before treatment, PC3 cells were seeded in 96‐well culture plates and allowed to achieve 60%–75% confluence. The cells were treated for 24 h with varied doses of PHN (1, 2.5, or 5 μM). The CCK8 solution (10 μL) was added to each well of the plate and incubated for 2 h. All tests were performed in triplicate, and the optical density (OD) was measured at 450 nm using a spectrophotometer (Sunrise‐Basic; Tecan Group, Ltd.).

### 2.4. MTT Assay

Cells (1 × 10^4^ cells/dL) were seeded in each well of 96‐well plates for 12 h and treated with various doses of PHN ranging from 0 μM to 5 μM for 24 h. After that, 10 μL of MTT solution was added into each well and incubated for another 6 h at 37°C. The formazan crystals were solubilized by dissolving in 100 μL of acidic isopropanol before recording absorbance readings. The absorbance of each well at 593 nm was measured using a spectrophotometer (Sunrise‐Basic; Tecan Group, Ltd.). The results were presented as a percentage of PHN‐untreated control cells, with absorbance at 655 nm used as control. All data were obtained from at least three trials.

### 2.5. Assessing Mitochondrial Biogenesis

A MitoBiogenesis In‐Cell ELISA Kit (Abcam, Cambridge, UK) was used to study the regulation of mitochondrial biogenesis. Cells (2 × 10^4^ cells/well) were seeded in a 96‐well plate and incubated overnight. Cells were fixed with 4% paraformaldehyde and washed with PBS. After that, 0.5% acetic acid was added to block endogenous alkaline phosphatase (AP) activity. Triton X‐100 (0.1%) was used to permeabilize the cells. Cells were treated with primary antibodies targeting mitochondrial DNA‐encoded cytochrome c oxidase subunit I (COX‐I) and nuclear DNA‐encoded succinate dehydrogenase complex subunit A (SDH‐A). COX‐I was detected using a horseradish peroxidase (HRP)‐labeled antibody; in contrast, SDH‐A was detected using an AP‐labeled antibody. The COX‐I/SDH‐A in‐cell ELISA reports the relative abundance of an mtDNA‐encoded subunit (COX‐I) to a nuclear‐encoded subunit (SDH‐A) as a proxy for mitochondrial biogenesis; signals were collected within the linear range and normalized across wells.

### 2.6. Western Blotting

A lysis solution was generated using protease (1% protease inhibitor cocktail [Sigma‐Aldrich, St. Louis, MO, USA]) and phosphatase inhibitors (PhosphoSafe Extraction Reagent [Novagen Inc., Madison, WI, USA]) to extract cytosolic proteins from cells. A BCA Assay Kit (Sigma‐Aldrich, St. Louis, MO, USA) determined the protein concentration. We used SDS–PAGE to separate the proteins by mass and then transferred the proteins onto polyvinylidene difluoride membranes (Millipore). After that, we incubated the membranes with specific primary antibodies against E‐cadherin (ab53033; Abcam, Cambridge, UK), α‐smooth muscle actin (α‐SMA) (ab5694; Abcam, Cambridge, UK), Snail (ab85931; Abcam, Cambridge, UK), Slug (sc‐166476; Santa Cruz Biotechnology, Inc.), AMPK (ab32047; Abcam, Cambridge, UK), NRF1 (ab34682; Abcam, Cambridge, UK), SIRT1 (ab110304; Abcam, Cambridge, UK), and β‐actin (A5441; Sigma‐Aldrich, St. Louis, MO, USA) at a dilution of 1:2000. Subsequently, secondary antibody incubation was done with matching HRP‐conjugated secondary antibodies, goat anti‐rabbit IgG HRP (ab6721; Abcam, Cambridge, UK), and antimouse IgG HRP (7076, Cell Signaling Biotechnology System, Beverly, MA) for 1 h at room temperature. The Western‐Ready ECL Substrate Plus Kit (426,316, BioLegend, San Diego, CA, USA) was used to detect immunoreactivity, and the MultiGel‐21 imaging system (Tangshan Top Biotechnology, Co., Ltd., Hebei, China) was used for visualization of the bands. Protein concentration was evaluated by developing a BSA standard curve, which was then applied to the cell lysates for analysis.

### 2.7. Immunofluorescence Staining

After pretreating PC3 cells with 0.1% Triton‐X 100 (Sigma‐Aldrich, St. Louis, MO, USA), they were blocked in 5% bovine serum (Sigma‐Aldrich, St. Louis, MO, USA) for immunocytochemical analysis. After placing them on two‐well chamber slides, incubation of primary antibodies with antibodies against E‐cadherin (1:100 in 1% bovine serum) and α‐SMA (1:100 in 1% bovine serum) was done overnight; in contrast, that for AMPK (in 1% bovine serum) was done for 1 h. Secondary antibody incubation was performed using antimouse/rabbit FITC‐conjugated antibodies (1:200 in 1% bovine serum; Molecular Probe). The nuclei were identified with 4,6‐diamidino‐2‐phenylindole (DAPI; Santa Cruz Biotechnology, USA). All images were acquired under identical magnification and microscopy conditions using an Olympus fluorescent microscope (CK41). The images were analyzed using the ipwin32 software (Image‐Pro Plus Version 6; Media Cybernetics, Inc.). Adjustments to brightness, contrast, and color balance were uniformly applied, and no nonlinear adjustments were made.

### 2.8. Wound Repair

A wound‐healing experiment was conducted using an Ibidi culture insert system (Applied Biophysics, Troy, NY, USA) to gauge the mobility of PC3 cells. PC3 cells were seeded in each compartment of the ibidi culture insert at 3.5 × 10^4^ cells/100 μL density. The inserts were removed after 24 h, and RPMI complete medium was added to the culture plate. A Leica AF 6000 LX microscope (Leica, Wetzlar, Germany) was used for live cell imaging to take pictures of cell migration immediately after incubation and 24 h later at 37°C. For each image, the Quantity One software (Version 4.6.6; Bio‐Rad Laboratories, Inc.) was used to calculate the distance between the edges of the gaps.

### 2.9. Statistical Analysis

Each experiment was performed at least thrice. Standard deviation (SD) was used to express the values in the bar graphs. For datasets with three or more groups, one‐way ANOVA followed by Tukey’s post hoc test was performed. The PRISM software was used for all statistical calculations. Statistical significance was set at *p* < 0.05.

## 3. Results

### 3.1. PHN Treatment Resulted in Modest Changes in PC3 Cell Viability and Proliferation Under the Tested Conditions

To evaluate the cellular responses of PC3 cells to PHN exposure, the CCK8 and MTT assays were used to assess cell viability and proliferation, respectively. This in vitro screening allowed us to evaluate the effects of PHN on PC3 cell proliferation and viability under noncytotoxic conditions. As shown in Figure 1(a), PHN treatment resulted in a slight decrease in cell viability, while overall cell survival remained above 90% across all tested concentrations. At 5 μM PHN, cell viability showed a modest reduction compared with the control group (approximately 98% vs. 90%, respectively). As depicted in Figure 1(b), PHN treatment was associated with a concentration‐dependent decrease in cell proliferation. The relative cell proliferation rate was approximately 95% in the control group and 79% in the group treated with 5 μM of PHN. A statistically significant difference in cell proliferation was observed at the 5 μM concentration. At 24 h, PHN treatment resulted in modest reduction in cell viability and proliferation, reaching approximately 90% and ∼79% of control at 5 μM, respectively (mean ± SD, *n* = 3).

FIGURE 1PHN inhibits PC3 cell viability and proliferation. These results showed the viability and proliferation of PC3 cells. (a) After treatment with 0, 1, 2.5, and 5 μM PHN for 24 h, the viability of PC3 cells was measured. (b) The proliferation rate measured by an MTT assay showed a similar result, indicating that PC3 cells were significantly inhibited by 5 μM PHN. Data shown as mean ± SD with individual data points (*n* = 3 independent experiments). One‐way ANOVA with Tukey post hoc unless otherwise indicated.

**Figure:**
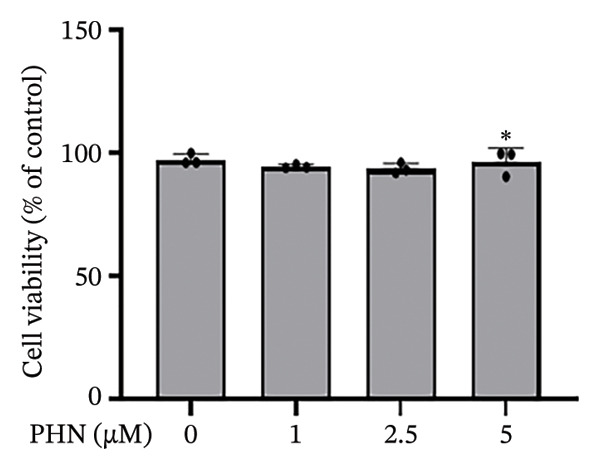
(a)

**Figure:**
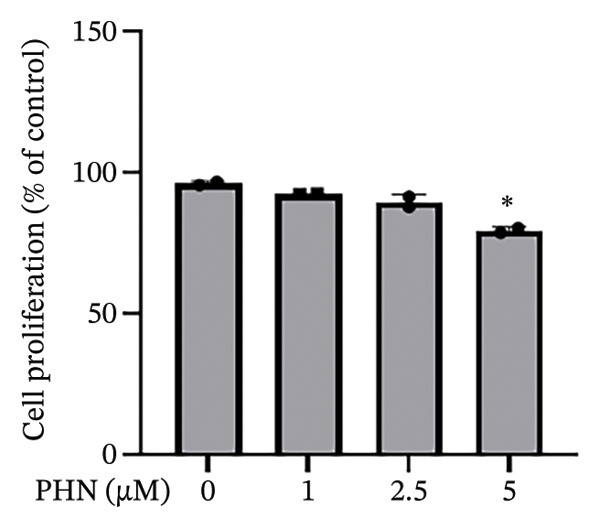
(b)

### 3.2. PHN Altered Mitochondrial‐Related Markers, Including the COX‐I/SDH‐A Ratio and the Expression of SIRT1 and NRF1

**FIGURE 2:**
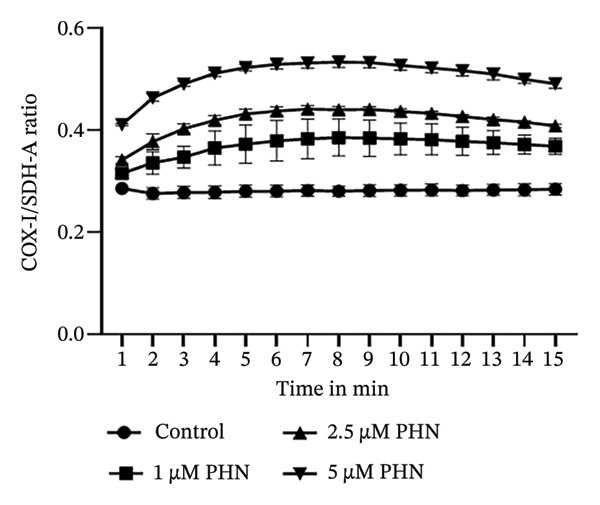
PHN activates mitochondrial biogenesis. PHN attenuates mitochondrial dysfunction of PC3 cells. The ratio of COX1/SDH‐A was utilized to indicate mitochondrial biogenesis. PHN attenuates mitochondrial dysfunction in PC3 cells.

We investigated the effects of PHN on mitochondrial‐related parameters in PC3 cells. We measured the ratio of mitochondria‐encoded COX1 proteins to nuclear DNA‐encoded SDH‐A proteins as an indirect indicator associated with mitochondrial content. As depicted in Figure 2, treatment with different concentrations of PHN resulted in an elevated COX1/SDH‐A ratio compared to control cells, suggesting alterations in mitochondrial‐related characteristics. PHN increased the COX‐I/SDH‐A signal ratio (in‐cell ELISA) and was associated with increased expression of SIRT1 and NRF1, which are proteins related to mitochondrial regulation.

We examined the protein expression levels of AMPK, SIRT1, and NRF1, which are proteins associated with mitochondrial regulation, to explore PHN‐related cellular responses. As shown in Figure 3(a), PHN treatment was associated with dose‐dependent changes in the expression levels of AMPK, SIRT1, and NRF1. Quantification of three independent Western blot experiments supported these observations. Figure 3(b) shows that the cells treated with higher concentrations of PHN exhibited increased protein expression levels compared with untreated control cells. Furthermore, immunofluorescence staining revealed increased intracellular AMPK protein immunoreactivity in PC3 cells treated with 5 μM of PHN compared to the control group (Figure 3(c)). These results indicate that PHN exposure is associated with changes in AMPK‐, SIRT1, and NRF1‐related protein expression, suggesting involvement of AMPK‐associated cellular responses rather than definitive regulation of mitochondrial biogenesis.

FIGURE 3Factors related to mitochondrial energy metabolism exhibit higher expression after PHN treatment. (a) Western blotting analysis of PC3 cells incubated with various concentrations of PHN (0 μM, 1 μM, 2.5 μM, and 5 μM) is shown as a representative result. Factors associated with mitochondrial biogenesis, such as AMPK, SIRT1, and NRF1, exhibited a dose‐dependent increase in expression following a 24‐h incubation with PHN. (b) We measured the levels of AMPK, SIRT1, and NRF1 proteins. Data are presented as means ± SD. ^∗^ *p* < 0.05 compared with control. (c) Immunofluorescence analysis of PC3 cells treated with PHN for 24 h shows the expression of AMPK. Compared to control cells, treatment with 5 μM of PHN significantly increased the fluorescence intensity of AMPK. Data are shown as mean ± SD with individual data points (*n* = 3 independent experiments). One‐way ANOVA with Tukey post hoc unless otherwise indicated.

**Figure:**
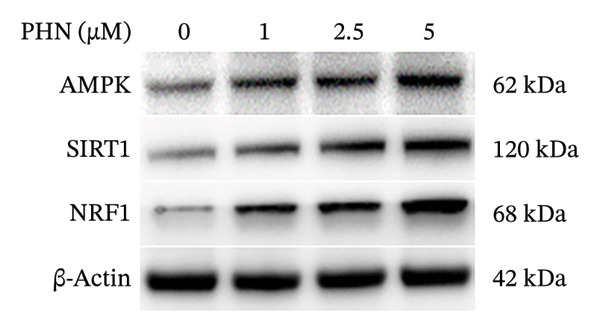
(a)

**Figure:**
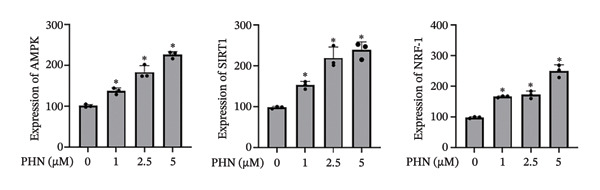
(b)

**Figure:**
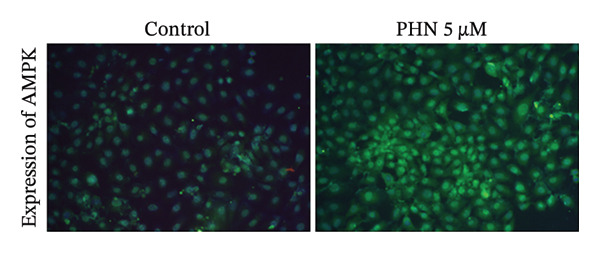
(c)

### 3.3. PHN Treatment Was Associated With Reduced Migratory Behavior of PC3 Cells

In the wound healing assay, PHN treatment was associated with reduced migration of PC3 cells into the wounded area (Figure 4(a)). PC3 cells in the control group migrated faster than those treated with PHN. In addition, cells treated with a high concentration of PHN exhibited reduced migratory capacity compared with those treated with lower concentrations. As shown in Figure 4(b), PHN treatment was associated with delayed wound closure in PC3 cells. Quantitative analysis showed that the remaining wound area was higher in PHN‐treated cells compared with untreated controls, with a dose‐dependent trend (*p* < 0.05). These data indicate that PHN treatment is associated with reduced PC3 cell migration in vitro under the experimental conditions used. PHN treatment was associated with decreased wound closure in a dose‐dependent manner; quantification based on migration distance (μm) or percentage of wound closure yielded comparable results (mean ± SD, *n* = 3).

FIGURE 4PHN inhibits PC3 cell migration. (a) In the wound healing experiment, the initial blank area of the control group at 0 h was presented as the control (100%). (b) Quantification of cell migration (% of wound closure relative to 0 h) after 24 h under different PHN concentrations. Values represent mean ± SD from three independent experiments. Data are shown as mean ± SD with individual data points (*n* = 3 independent experiments). One‐way ANOVA with Tukey post hoc unless otherwise indicated.

**Figure:**
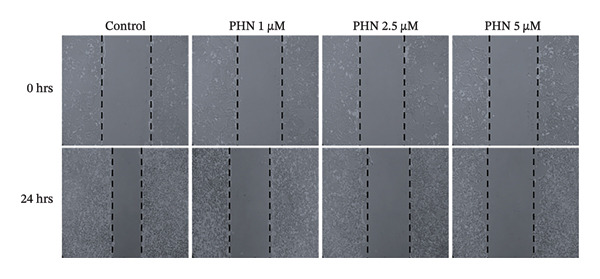
(a)

**Figure:**
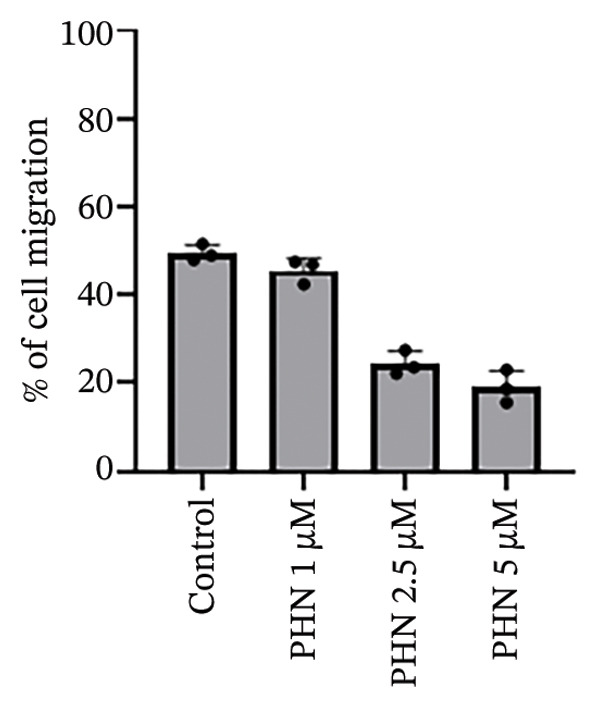
(b)

EMT and cell migration are closely associated processes involved in tumor progression and metastasis. Therefore, following the evaluation of cell migratory behavior, we examined the expression of EMT‐associated markers in PC3 cells following PHN treatment. Specifically, we assessed the epithelial marker E‐cadherin and mesenchymal‐associated markers, including α‐SMA, Snail, and Slug. The control group consisted of cells cultured under identical conditions without PHN treatment. Our findings showed that PHN treatment was associated with increased E‐cadherin expression and decreased α‐SMA, Snail, and Slug expression in a concentration‐dependent manner (Figure 5(a)). Quantitative analysis demonstrated an increase in E‐cadherin expression and a decrease in α‐SMA, Snail, and Slug expressions in PHN‐treated cells compared with control cells (Figure 5(b)). Immunofluorescence staining revealed intracellular immunoreactivity of E‐cadherin and α‐SMA. Additionally, compared with untreated cells, cells treated with 5 μM of PHN exhibited increased E‐cadherin immunoreactivity and reduced α‐SMA immunoreactivity (Figure 5(c)). These results suggested that PHN treatment is associated with changes in EMT‐related marker expression in PC3 cells under the experimental conditions used.

FIGURE 5PHN inhibits the EMT processes in PC3 cells. (a) After exposure to various concentrations of PHN (0 μM, 1 μM, 2.5 μM, and 5 μM), the levels of PC3 cells’ E‐cadherin, α‐SMA, Snail, and Slug proteins were evaluated. Loading control was β‐actin. (b) Quantification graphs display the levels of EMT‐related proteins following treatment with different concentrations of PHN. (c) PC3 cells were cultured with or without 5 μM of PHN and stained with antibodies against E‐cadherin and α‐SMA. Data are shown as mean ± SD with individual data points (*n* = 3 independent experiments). One‐way ANOVA with Tukey post hoc unless otherwise indicated. Panels derive from independent membranes; quantification is within‐blot after β‐actin normalization; cross‐panel band intensities are not directly comparable.

**Figure:**
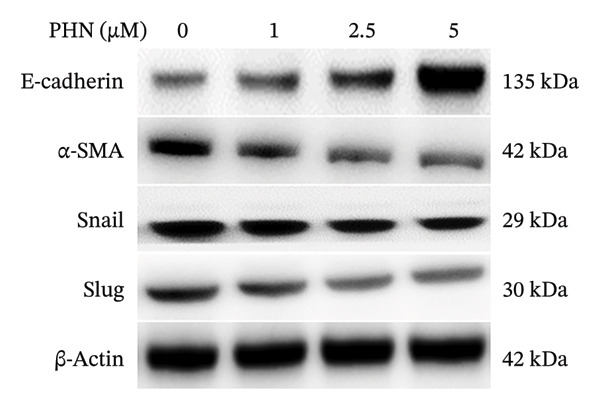
(a)

**Figure:**
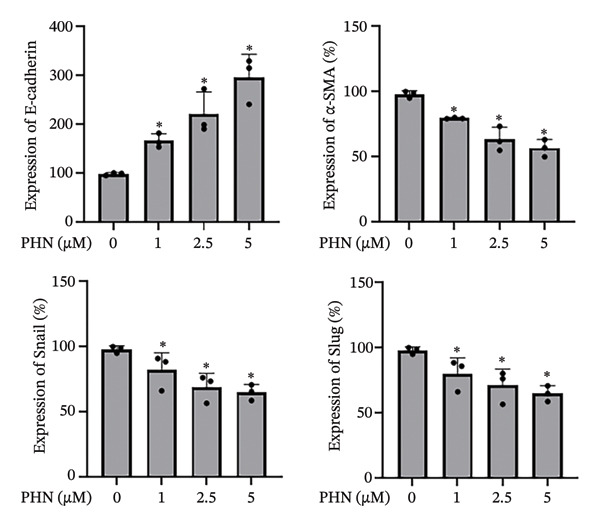
(b)

**Figure:**
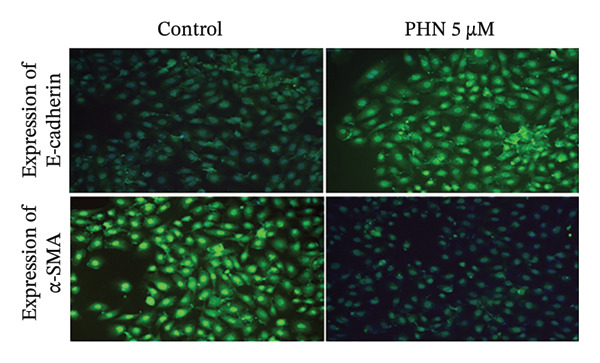
(c)

### 3.4. Effect of PHN on EMT‐Related Marker Expressions Was Modulated by AMPK Inhibitors

To further explore the involvement of AMPK‐associated responses, we examined whether the effects of PHN on EMT‐related marker expression were altered by pharmacological inhibition of AMPK in PC3 cells. We assessed the expression of EMT‐related markers, in the presence or absence of an AMPK inhibitor. Comp C, a pharmacological inhibitor commonly used to interfere with AMPK activity, was added to PC3 cells treated with either 0 μM or 5 μM PHN. Figure 6(a) presents the results of Western blotting, and Figure 6(b) shows the corresponding quantitative analysis. Consistent with previous observations, PHN treatment was associated with altered expression of EMT‐related markers in PC3 cells compared with the untreated control group. No significant differences in EMT‐related marker expression were observed in cells treated with Comp C alone compared with control cells. However, the PHN‐associated increase in E‐cadherin expression was attenuated in the presence of Comp C. In the presence of Comp C, E‐cadherin expression levels in PHN‐treated PC3 cells were reduced compared with cells treated with PHN alone. The expression levels of α‐SMA, Snail, and Slug, which were decreased following PHN treatment, were increased upon co‐treatment with Comp C. Collectively, these results indicate that the PHN‐associated changes in EMT‐related marker expression were modified in the presence of Comp C after 24 h of treatment. These findings suggest that PHN‐associated changes in EMT‐related marker expression are pharmacologically sensitive to AMPK inhibition, supporting an association with AMPK‐related cellular responses rather than definitive pathway regulation.

FIGURE 6Effects of EMT downregulation are reversed by AMPK inhibitors. Effect of an AMPK inhibitor, Comp C, on EMT‐related protein levels in PC3 cells. (a) Western blotting was performed to analyze the effect of an AMPK inhibitor, Comp C, on the protein expression of EMT‐related factors in PC3 cells. (b) Quantification of the protein expression levels of EMT‐related factors following treatment with PHN (0 μM or 5 μM) or Comp C (0 μM or 10 μM). Data are presented as means ± SD. ^∗^ *p* < 0.05 versus group without PHN or Comp C treatment; ^#^ *p* < 0.05 versus treatment with PHN alone (mean ± SD, *n* = 3 independent experiments; individual data points shown). Panels derive from independent membranes; quantification is within‐blot after β‐actin normalization; cross‐panel band intensities are not directly comparable.

**Figure:**
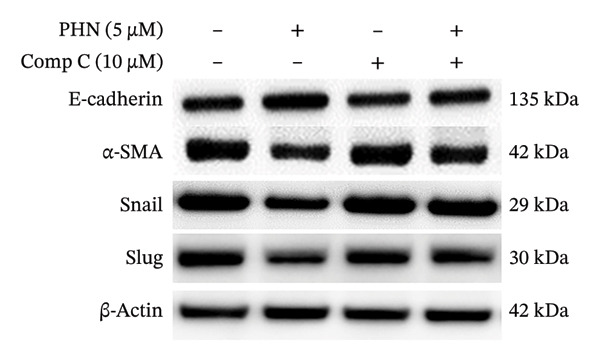
(a)

**Figure:**
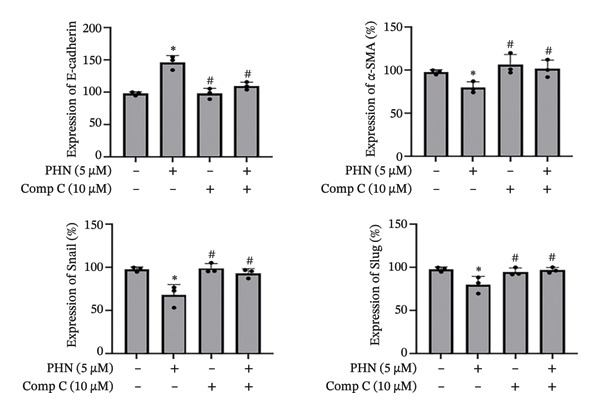
(b)

### 3.5. PHN and the AMPK Activator AICAR are Associated With Changes in EMT‐Related Protein Expression

To further explore whether PHN‐associated changes in EMT‐related marker expression were related to AMPK‐associated responses, AICAR, a pharmacological activator commonly used to stimulate AMPK signaling, was applied alone or in combination with PHN. As shown in Figure 7(a), AICAR treatment was associated with increased expression of AMPK, SIRT1, NRF1, and E‐cadherin levels, along with decreased expression of α‐SMA, Snail, and Slug. When combined with PHN (2.5 μM), AICAR produced changes in AMPK and E‐cadherin expression that were comparable to those observed with either treatment alone, without evidence of synergistic enhancement. Quantitative analysis (Figure 7(b)) showed that AICAR treatment resulted in EMT‐related marker expression patterns consistent with those observed following PHN treatment, while co‐treatment with Comp C altered these patterns. These findings support an association between PHN exposure and AMPK‐related cellular responses in the regulation of EMT‐related marker expression, without demonstrating the necessity or sufficiency of AMPK activation.

FIGURE 7AICAR activates AMPK and cooperates with low‐dose PHN to modulate EMT in PC3 cells. (a) Representative immunoblots for AMPK, SIRT1, NRF1, E‐cadherin, α‐SMA, Snail, and Slug in PC3 cells treated for 24 h under the following conditions: control, PHN 2.5 μM, PHN 2.5 μM + Compound C 10 μM, and PHN 2.5 μM + AICAR 0.5 mM. β‐Actin served as a loading control. (b) Densitometric quantification normalized to β‐actin (mean ± SD, *n* = 3 independent experiments; individual data points shown). One‐way ANOVA with Tukey’s post hoc test; ^∗^ *p* < 0.05 versus Vehicle; ^#^ *p* < 0.05 versus the corresponding single‐agent condition.

**Figure:**
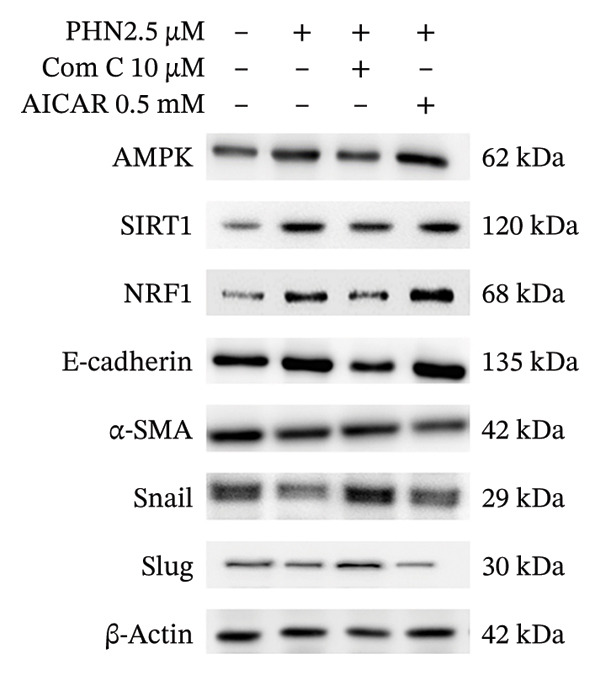
(a)

**Figure:**
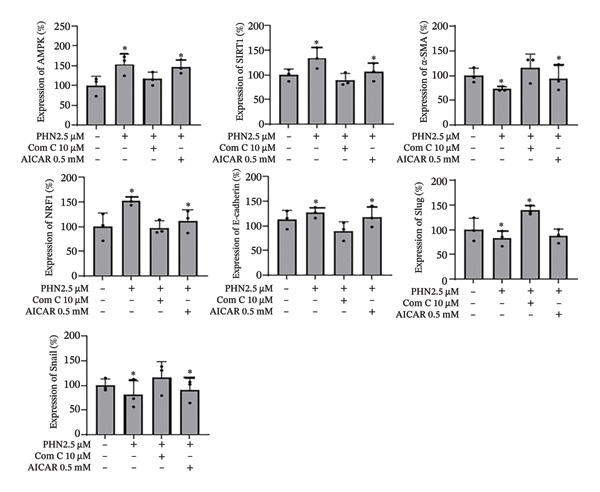
(b)

### 3.6. AMPK‐Associated Pharmacological Modulation Influences PHN‐Related Effects on PC3 Cell Migration

To explore whether PHN‐associated changes in PC3 cell migration were related to AMPK‐associated responses, wound‐healing assays were performed in the presence of AICAR or Comp C. As shown in Figure 8(a), PHN (5 μM) was associated with reduced wound closure compared with the control group, while this effect was attenuated in the presence of Comp C. Conversely, AICAR treatment produced wound closure patterns comparable to those observed following PHN treatment, without evidence of enhanced inhibition upon co‐treatment. Quantification of migration rates (Figure 8(b)) showed that AICAR treatment was associated with reduced wound closure compared with the control group (*p* < 0.05), whereas co‐treatment with Comp C altered the wound closure pattern observed with PHN alone. These results suggest that PHN‐associated changes in PC3 cell migration are pharmacologically sensitive to AMPK modulation, supporting an association with AMPK‐related cellular responses rather than definitive AMPK activation.

FIGURE 8AICAR and Compound C verify AMPK‐dependent regulation of cell migration. (a) Representative wound‐healing images at 0 h and 24 h for control, PHN 5 μM, PHN 5 μM + Compound C 10 μM, and PHN 5 μM + AICAR 0.5 mM. (b) Cell migration (%) computed from wound closure relative to 0 h (mean ± SD, *n* = 3; individual data points shown). One‐way ANOVA with Tukey’s test; ^∗^ *p* < 0.05 versus control; ^#^ *p* < 0.05 versus PHN 5 μM.

**Figure:**
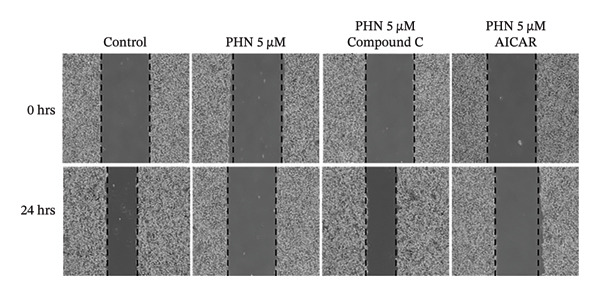
(a)

**Figure:**
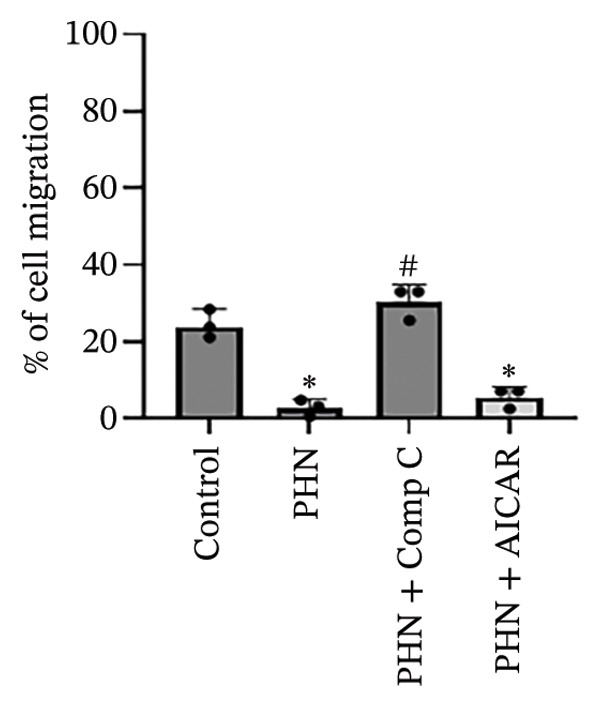
(b)

## 4. Discussion

ADT is the first‐line systemic treatment for metastatic PCa. However, PCa often progresses to castration‐resistance PCa even after administering various therapies [27]. Therefore, there is a continued need to identify novel therapeutic agents with alternative cellular targets or mechanisms of action. In this study, PC3 cells were used as a commonly employed in vitro model of aggressive, androgen‐independent PCa. Alterations in mitochondrial function and mitochondrial‐related pathways have been implicated in tumor progression and therapeutic responses in several cancer types [15, 28, 29]. PHN has been reported to influence mitochondrial‐related processes in different experimental contexts [12–14]. However, the effects of PHN on mitochondrial‐related cellular responses in PCa cells have not been previously investigated. In the present study, PHN treatment was associated with changes in mitochondrial‐related markers and cellular behaviors, while exerting minimal effects on cell viability under the tested conditions.

EMT is a cellular process associated with enhanced migratory capacity and metastatic potential of tumor cells. During EMT, tumor cells often exhibit reduced epithelial characteristics, such as decreased E‐cadherin expression, along with increased expression of mesenchymal‐associated markers, including α‐SMA, Snail, and Slug. Modulation of EMT‐related processes has been reported to influence PCa progression and migratory behavior [30, 31]. Although no study has directly examined the relationship between PHN and EMT in PCa, indirect evidence suggests potential associations. AMPK signaling has been implicated in the regulation of EMT‐related cellular processes. Previous studies have reported that PHN can influence cellular responses in different cell types through AMPK‐related signaling pathways [9–11]. In the present study, PHN treatment was associated with reduced migratory behavior of PCa cells in a dose‐dependent manner. PHN treatment was associated with increased expression of epithelial‐associated markers. Although EMT‐related pathways have been explored as potential targets in PCa, this study provides initial evidence that PHN is associated with alterations in EMT‐related marker expression and migratory behavior in PCa cells.

Only a few studies have evaluated the biological effects of PHN in cancer‐related models. Regarding potential mechanisms, Wang et al. investigated the effect of PHN on laryngeal squamous cell carcinoma and found that PHN‐associated autophagy may involve the AMPK/mTOR/p70S6K pathway [9]. Mechanisms underlying the effects of PHN in PCa cells remain incompletely understood. Our results showed that PHN treatment was associated with reduced migratory behavior of PC3 cells and altered expression of EMT‐related markers in a dose‐dependent manner. In the presence of Comp C, the PHN‐associated changes in EMT‐related marker expression were modified. This observation suggests that PHN‐associated changes in PCa cell migration may be pharmacologically sensitive to AMPK‐related signaling, without establishing a definitive regulatory pathway. While our data indicate changes in biogenesis‐related markers, additional assays, such as mtDNA copy number, TFAM/PGC‐1α expression, or citrate synthase activity, would be required to confirm mitochondrial biogenesis. EMT‐related processes in PCa cells may also be influenced by PHN through alternative signaling pathways. PHN has been reported to modulate the TGF‐β1/Smad2/3 signaling pathway in hepatic cells, leading to altered inflammatory responses [8]. In PCa cells, AMPK signaling has been implicated in the regulation of cell migration and EMT‐related processes through the TGF‐β/SMAD signaling pathway [32]. However, the relationship between PHN and SMAD in PCa merits further investigation.

This study has several limitations that warrant consideration, including the use of a single PC3 cell line. Other PCa cell lines representing different molecular or androgen‐dependence statuses were not examined. Additional limitations include the exclusive use of in vitro cell‐based models without validation in tissue samples, as well as the absence of experiments evaluating the effects of PHN in combination with hormone‐based therapies. Therefore, further studies using additional cellular models, in vivo systems, and clinical samples are required to clarify the relevance of PHN‐associated cellular responses in PCa and to explore its potential translational implications.

In conclusion, this study provides initial evidence that PHN exposure is associated with changes in cellular behaviors and molecular marker expression in PC3 PCa cells under non‐cytotoxic conditions. Specifically, PHN treatment was associated with altered migratory behavior and changes in EMT‐related and AMPK‐associated protein expression. While pharmacological modulation of AMPK influenced these PHN‐associated responses, the present findings do not establish definitive pathway regulation or therapeutic efficacy. Together, these results support the relevance of PHN as a compound of interest for further investigation into its cellular effects in PCa models, warranting additional studies using complementary experimental systems.

## 5. Conclusion

PHN exposure was associated with modest changes in cellular behavior and molecular marker expression in PC3 PCa cells under non‐cytotoxic conditions. Specifically, PHN treatment was associated with altered migratory behavior and changes in EMT‐related and mitochondrial‐associated proteins, including AMPK‐, SIRT1‐, and NRF1‐related responses. While pharmacological modulation of AMPK influenced these PHN‐associated effects, the present findings do not demonstrate definitive activation of mitochondrial biogenesis or causal pathway regulation.

## Author Contributions

Cheng‐Hsin Lu, Chun‐Hsien Wu, Victor C. Lin, and Chiang‐Ting Wang designed and conceived the study; Pei‐Fang Hsieh, Hsing‐Chia Mai, Wei‐Lun Huang, and Sih‐Han Chen performed the experiments; Richard Chen‐Yu Wu and Chien‐Ming Lai performed the statistical analysis; Cheng‐Hsin Lu, Chun‐Hsien Wu, Victor C. Lin, and Chiang‐Ting Wang drafted the article and made critical revisions to the manuscript.

## Funding

This work was supported by Kaohsiung Armed Forces General Hospital Research (grant no. KAFGH_D_109026).

## Disclosure

We have disclosed funding support from Kaohsiung Armed Forces General Hospital; however, this does not influence the objectivity of the research. All authors have read and approved the final manuscript.

## Ethics Statement

The authors have nothing to report.

## Conflicts of Interest

The authors declare no conflicts of interest.

## Data Availability

The datasets generated and analyzed during this study are available from the corresponding author upon reasonable request.
